# CaMKKβ Is Involved in AMP-Activated Protein Kinase Activation by Baicalin in LKB1 Deficient Cell Lines

**DOI:** 10.1371/journal.pone.0047900

**Published:** 2012-10-22

**Authors:** Ying Ma, Fuzhen Yang, Ying Wang, Zhiyan Du, Daihua Liu, Hongxia Guo, Jingkang Shen, Hongli Peng

**Affiliations:** State Key Laboratory of Drug Research, Shanghai Institute of Materia Medica, Chinese Academy of Sciences, Shanghai, PR China; Boston University School of Medicine, United States of America

## Abstract

AMP-activated protein kinase (AMPK) plays an important role in mediating energy metabolism and is controlled mainly by two upstream kinases, LKB1 or Ca^2+^/calmodulin-dependent protein kinase kinase-β (CaMKKβ). Previously, we found that baicalin, one of the major flavonoids in a traditional Chinese herb medicine, *Scutellaria baicalensis*, protects against the development of hepatic steatosis in rats feeding with a high-fat diet by the activation of AMPK, but, the underlying mechanism for AMPK activation is unknown. Here we show that in two LKB1-deficient cells, HeLa and A549 cells, baicalin activates AMPK by α Thr-172 phosphorylation and subsequent phosphorylation of its downstream target, acetyl CoA carboxylase, at Ser-79, to a similar degree as does in HepG2 cells (that express LKB1). Pharmacologic inhibition of CaMKKβ by its selective inhibitor STO-609 markedly inhibits baicalin-induced AMPK activation in both HeLa and HepG2 cells, indicating that CaMKKβ is the responsible AMPK kinase. We also show that treatment of baicalin causes a larger increase in intracellular Ca^2+^ concentration ([Ca^2+^]_i_), although the maximal level of [Ca^2+^]_i_ is lower in HepG2 cells compared to HeLa cells. Chelation of intracellular free Ca^2+^ by EDTA and EGTA, or depletion of intracellular Ca^2+^ stores by the endoplasmic reticulum Ca^2+^-ATPase inhibitor thapsigargin abrogates baicalin-induced activation of AMPK in HeLa cells. Neither cellular ATP nor the production of reactive oxygen species is altered by baicalin. Finally, in HeLa cells, baicalin treatment no longer decreases intracellular lipid accumulation caused by oleic acid after inhibition of CaMKKβ by STO-609. These results demonstrate that a potential Ca^2+^/CaMKKβ dependent pathway is involved in the activation of AMPK by baicalin and suggest that CaMKKβ likely acts as an upstream kinase of AMPK in response to baicalin.

## Introduction

The AMP-activated protein kinase (AMPK) is a critical regulator of energy homeostasis, and is a potential target for treatment of metabolic diseases as well as cancer. AMPK is a highly conserved serine/threonine protein kinase composed of an α catalytic subunit and β and γ regulatory subunits and acts as a “master switch” for lipid metabolism (for more detail see review: [1]). AMPK is activated allosterically by alterations in the intracellular AMP:ATP ratio that occurs in response to energetic stress and requires phosphorylation of Thr-172 in the activation loop of the catalytic α subunit [2]. Activation of AMPK switches off fatty acid synthesis and switches on fatty acid oxidation by phosphorylation and inactivation of acetyl-CoA carboxylase (ACC), the rate-limiting enzyme of fatty acid synthesis. AMPK is also activated by a number of pathological stresses, including hypoxia, oxidative stress, exercise and dietary hormones, such as leptin and adiponectin [1]. Due to its important role in regulation of energy homeostasis, AMPK is a promising drug target for the treatment of type 2 diabetes and other diseases of the metabolic syndrome [3]. In addition, AMPK has recently been implicated in the regulation of cell growth, protein synthesis and cell cycle, which are all processes important for tumor formation, making AMPK a potential target for cancer therapy [4].

A large body of evidence demonstrates that dysfunction of hepatic AMPK represents a key mechanism for hepatic lipid accumulation and hyperlipidemia [5], [6], [7], [8], [9]. Metformin (an anti-diabetic drug) and polyphenols, such as resveratrol from red grapes [10] and theaflavins from black tea [5], have been shown to decrease hepatic fat and improve fatty liver disease via activation of AMPK. Our previous works [11] also revealed that in a rat model of the high-fat diet (HFD) feeding for obesity and hepatic steatosis baicalin stimulates AMPK activity by enhancing its phosphorylation in liver and skeleton muscle and increases phosphorylation of the AMPK downstream target ACC, thereby effectively reduces hepatic lipid accumulation, which in turn attenuates metabolic disorders and hepatic steatosis in obese rats. Additionally, we observed a similar phenomenon with the structurally related flavone, luteolin, which is also able to activate AMPK and reduce enhanced intracellular lipid accumulation caused by palmitate in HepG2 hepatoma cells [12]. These findings suggest that AMPK has emerged as a critical mechanism for beneficial effects of natural polyphenols including baicalin on lipid metabolic disorders in type 2 diabetes and obesity-related fatty liver diseases. However, the molecular mechanisms that mediate baicalin-induced AMPK activation are poorly understood.

So far, at least two upstream kinases have been identified as activators of AMPK, the tumor suppressor LKB1 [13], [14] and Ca^2+^/calmodulin-dependent protein kinase kinase β (CaMKKβ) [15], [16], [17]. LKB1 has been considered as a constitutively active serine/threonine protein kinase that is ubiquitously expressed in mammalian cells and phosphorylates the catalytic α subunit of AMPK by an increase in the AMP:ATP ratio in cells [13], [14]. In contrast to LKB1, the activation of AMPK by CaMKKβ is initiated by an increase in intracellular Ca^2+^ and does not respond to change in the ATP:AMP ratio [15], [16], [17]. The discovery of AMPK as a substrate for CaMKKβ indicates that in addition to an increase of the AMP:ATP ratio, AMPK may also be regulated by a rise in intracellular Ca^2+^ concentration ([Ca^2+^]_i_) in response to physiological stimulation, or certain drugs.

Studies on how polyphenols, such as resveratrol, theaflavins and apigenin, activate AMPK have led to different mechanisms. A number of studies demonstrate that LKB1 may act as an upstream kinase responsible for resveratrol- and theaflavins-induced AMPK activation [5], [18], [19]. However, resveratrol has been recently reported to increase [Ca^2+^]_i_ through increased cAMP levels and activation of CaMKKβ, which phosphorylates and activates AMPK [20]. In addition, studies with apigenin showed that the Ca^2+^/CaMKKβ pathway is essential for the AMPK activation of apigenin [21]. Baicalin has been shown to activate cAMP signaling in rat mesenteric arteries [22] and to increase [Ca^2+^]_i_ in human leukemia HL-60 cells [23]. Baicalein, the aglycone of baicalin, has also been shown to increase [Ca^2+^]_i_ in human breast cancer cells [24] and human intestinal T84 cells [25]. Our previous studies on the anti-proliferative activity of novel baicalein derivatives synthesized by our lab [26] demonstrated that these compounds enhanced AMPK phosphorylation in LKB1-deficient HeLa cells and induced apoptosis. Therefore, we hypothesized if CaMKKβ is involved in the effect of baicalin on AMPK. Here we show that upon baicalin stimulation, AMPK activity increases multiple folds in two LKB1-deficient cells, HeLa and A549 cells, which is similar as does in HepG2 cells (that express LKB1), implying that CaMKKβ could be the responsible AMPK kinase. Several lines of evidence support this hypothesis. Firstly, pharmacological inhibition of CaMKKβ by its selective inhibitor STO-609 markedly inhibits baicalin-induced AMPK activity either in HeLa cells or in HepG2 cells. Secondly, baicalin increases [Ca^2+^]_i_, leading to activation of AMPK. In addition, baicalin also increases the phosphorylation of calmodulin-dependent protein kinase I (CaMKI), a substrate of CaMKKβ, which can blocked by STO-609. Finally, in HeLa cells, baicalin treatment no longer decreases intracellular lipid accumulation caused by oleic acid after inhibition of CaMKKβ by STO-609. Thus, CaMKKβ likely acts as an upstream kinase of AMPK in response to baicalin.

## Results

### Baicalin Stimulates AMPK Activity in LKB1-deficient Cells

**Figure 1:**
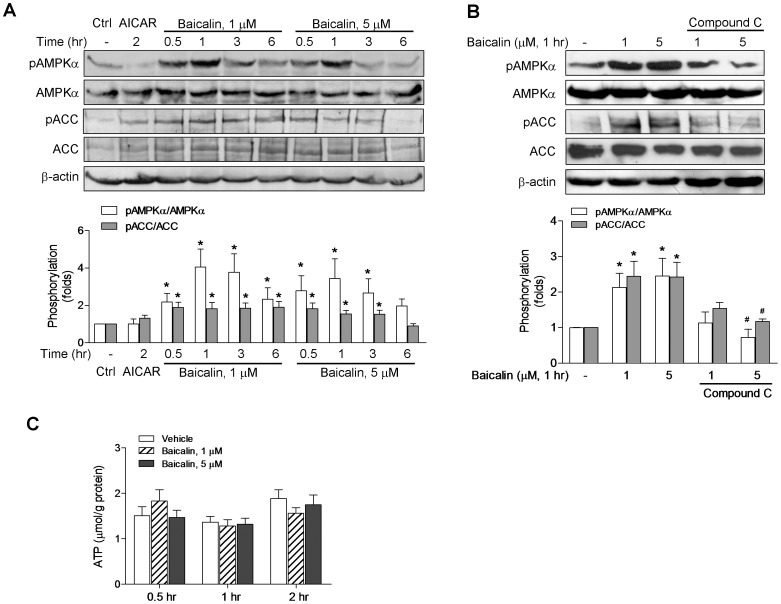
Baicalin increases the phosphorylation of AMPKα and ACC without effect on ATP in HeLa cells. Levels of AMPKα phosphorylated at Thr-172 (pAMPKα) and of AMPK substrate ACC phosphorylated at Ser-79 (pACC) as well as total AMPKα and ACC were determined by Western blotting. Cells were treated with baicalin or AICAR (500 µM) for the indicated times without (A) or with (B) pre-treatment of the AMPK inhibitor compound C (40 µM). Histograms represent the fold change in the pAMPKα/AMPKα or pACC/ACC ratio from at least three independent experiments. (C) Cells were incubated with baicalin for indicated times and ATP levels were measured. All values are the mean ± SE for three independent experiments. * *p*<0.05 compared to respective control; # *p*<0.05 compared to respective baicalin group in the absence of compound C. Representative Western blots are shown.

LKB1 and CaMKK are two upstream AMPKKs that phosphorylate and activate AMPK. Activation of AMPK via energy depletion is dependent on LKB1 [13], [14], [27], and activation of AMPK via increased intracellular Ca^2+^ is mainly dependent on CaMKKβ [15], [16], [17]. Since baicalin has been shown to increase [Ca^2+^]_i_ in human leukemia HL-60 cells [23] and some of novel baicalein derivatives synthesized by our lab [26] have been found to enhance AMPK phosphorylation and induce apoptosis in LKB1-deficient HeLa cells, we asked whether baicalin-stimulated AMPK signaling could be regulated by the upstream kinase CaMKKβ. Two cell lines deficient in LKB1, HeLa and A549 cells, were employed to study baicalin-induced AMPK activation. As the activation of AMPK correlates tightly with phosphorylation at Thr-172 (pAMPKα), we assessed the activation of AMPK by determining phosphorylation AMPKα and its primary downstream targeting enzyme, ACC, using immunoblots with specific phospho-Thr-172 and phospho-Ser-79 antibodies. After incubation with baicalin (1 and 5 µM), a time-dependent increase in phosphorylation of AMPKα was observed (Figure 1A). However, 500 µM AICAR, an AMP analogue activating AMPK through AMP- and LKB1-dependent mechanism [14], [28], had no effect on AMPKα phosphorylations in HeLa cells (Figure 1A). The marked phosphorylation of ACC paralleled the increase in AMPKα phosphorylation as shown in Figure 1A and B, indicating that baicalin activated AMPK leading to phosphorylation of its downstream target. The phosphorylation of AMPKα and ACC occurred and reached maximal levels within 1 h (Figure 1A). The increased phosphorylation of AMPKα and ACC caused by baicalin was blocked by the selective inhibitor of AMPK, compound C (Figure 1B), which has been used widely to evaluate the roles that AMPK plays in various cellular processes [9], [29].

**Figure 2:**
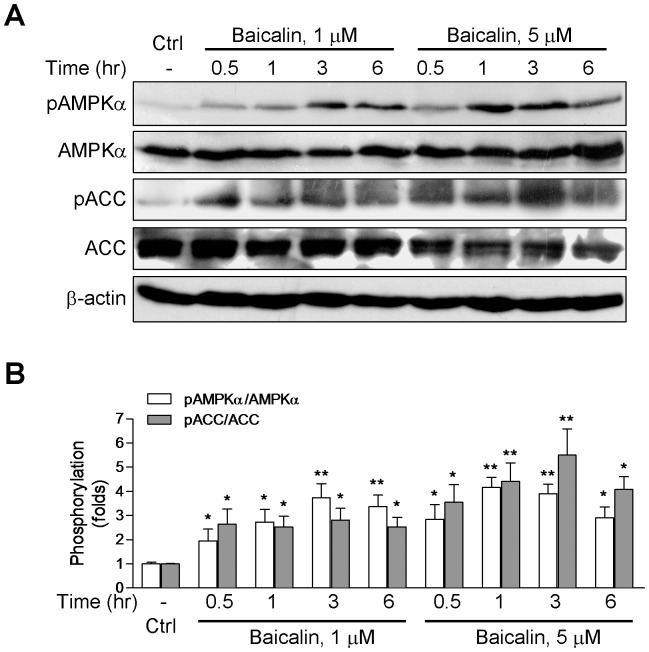
Baicalin increases the phosphorylation of AMPKα and ACC in A549 cells. (A) Representative Western blots for the phosphorylation of AMPKα at Thr-172 (pAMPKα) and the phosphorylation of ACC at Ser-79 (pACC) after treatment with baicalin for the indicated times. (B) Histogram represents the fold change in the pAMPKα/AMPKα or pACC/ACC ratio from at least three independent experiments. * *p*<0.05 and ** *p*<0.01 compared to respective control.

In studies carried out with A549 cells, baicalin increased the phosphorylation of AMPKα and ACC (Figure 2) to a similar extent compared with its effect in HeLa cells. These results provide direct evidence that baicalin could activate AMPK in the absence of LKB1, implying that there might be LKB1-independent mechanism mediating AMPK activation by baicalin.

Since AMPK is the intracellular energy sensor and a decrease in the cellular ATP can lead to AMPK activation, we measured the intracellular ATP concentrations along with AMPK and ACC phosphorylation. Treatment of HeLa cells with 1 and 5 µM baicalin for 0.5, 1 and 2 hr, respectively, did not produce obviously change in cellular ATP levels as shown in Figure 1C.

### Role of a Ca^2+^- and CaMKKβ-mediated Pathway in Baicalin-mediated AMPK Activation in the Absence of LKB1

**Figure 3:**
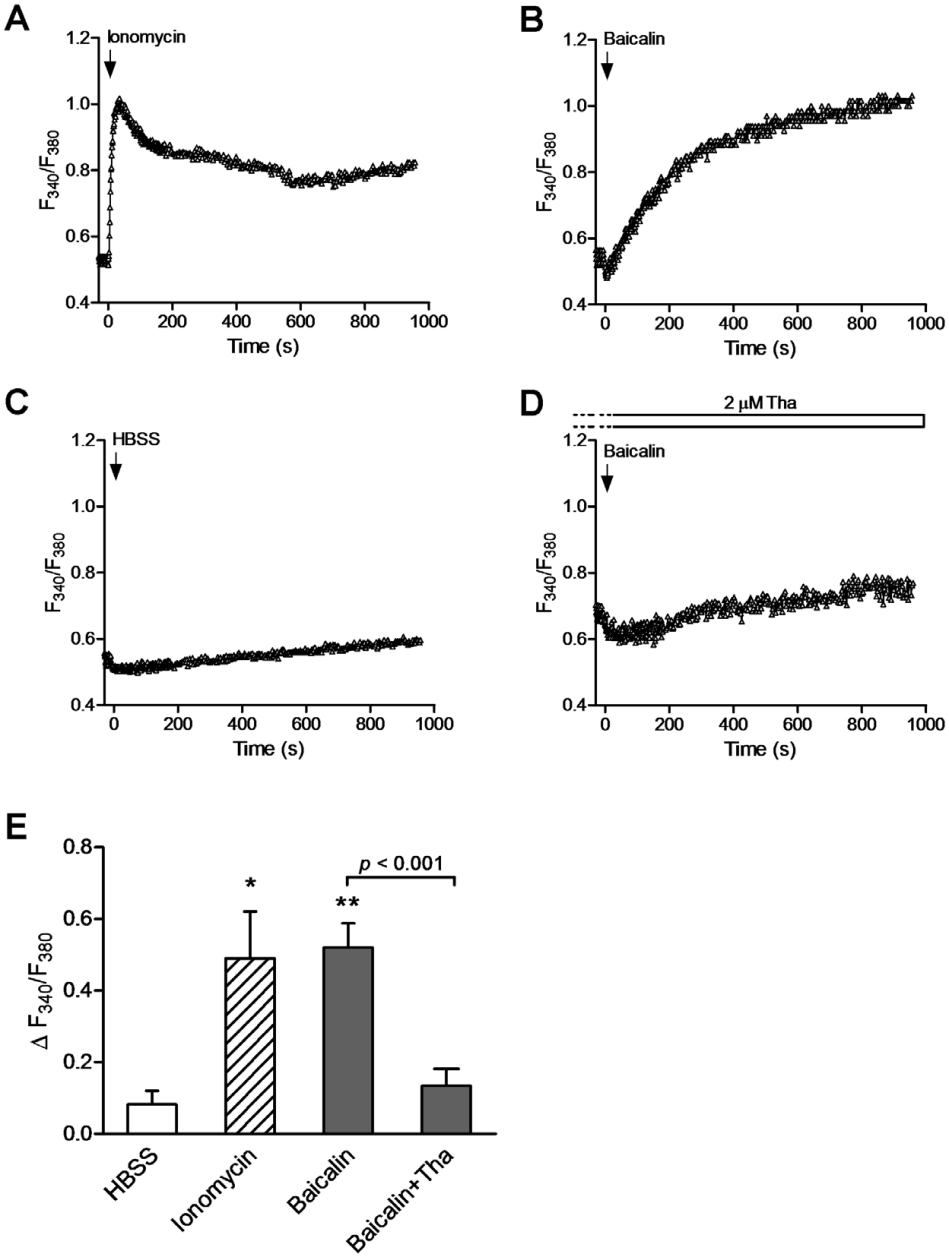
Baicalin increases the intracellular Ca^2+^ levels in HeLa cells. Traces show the increases in intracellular Ca^2+^ levels in response to application of ionomycin (1 µM) (A), baicalin (5 µM) in HBSS solution (B) and after depletion of intracellular Ca^2+^ stores by thapsigargin (Tha, D), as well as application of HBSS solution (C) in HeLa cells loaded with Ca^2+^ indicator fura 2-AM. Intracellular Ca^2+^ levels were estimated as the ratio of the signals (F_340_/F_380_). (E) Histogram summarizes the changes of intracellular Ca^2+^ levels (ΔF_340_/F_380_) measured after application of HBSS (open bars, n = 3), ionomycin (peak values, hatched bars, n = 3), baicalin in HBSS (solid bars, n = 7) or in the presence of Tha (solid bars, n = 3). * *p*<0.05 and ** *p*<0.01 compared to HBSS control; *p*<0.001 compared between two groups as indicated.

Given that baicalin-mediated activation of AMPK did not require LKB1, we explored the role of CaMKKβ. Since CaMKKβ responds to increased [Ca^2+^]_i_ and activates AMPK in an AMP-independent manner [15], [16], [17], we measured [Ca^2+^]_i_ after treatment of HeLa cells with baicalin or the Ca^2+^ ionophore ionomycin by using fura-2/AM. As exemplified in Figure 3A–C, administration of baicalin caused a slow increase in [Ca^2+^]_i_ reaching steady-state condition within 1 hr, while ionomycin evoked a rapid transient peak [Ca^2+^]_i_ within 30 sec followed by a sustained increase in [Ca^2+^]_i_. However, application of HBSS solution under identical conditions where both baicalin and ionomycin caused a larger increase in [Ca^2+^]_i_ (Figure 3A, B and E) induced a small increase in [Ca^2+^]_i_ (Figure 3C and E). Baicalin- and ionomycin-induced changes of [Ca^2+^]_i_ (ΔF_340_/F_380_) were 0.52±0.06 and 0.49±0.14 (peak values), respectively, which were significantly larger than that induced by HBSS (0.08±0.04, Figure 3E, *p*<0.05). The baicalin-evoked increases in [Ca^2+^]_i_ was due to the Ca^2+^ release from the intracellular Ca^2+^ stores, since the elevated [Ca^2+^]_i_ disappeared in the presence of 2 µM of the ER Ca^2+^-ATPase inhibitor thapsigargin (Figure 3D and E; ΔF_340_/F_380_∶0.13±0.05, *p*<0.001 compared with the values evoked by baicalin in HBSS solution).

**Figure 4:**
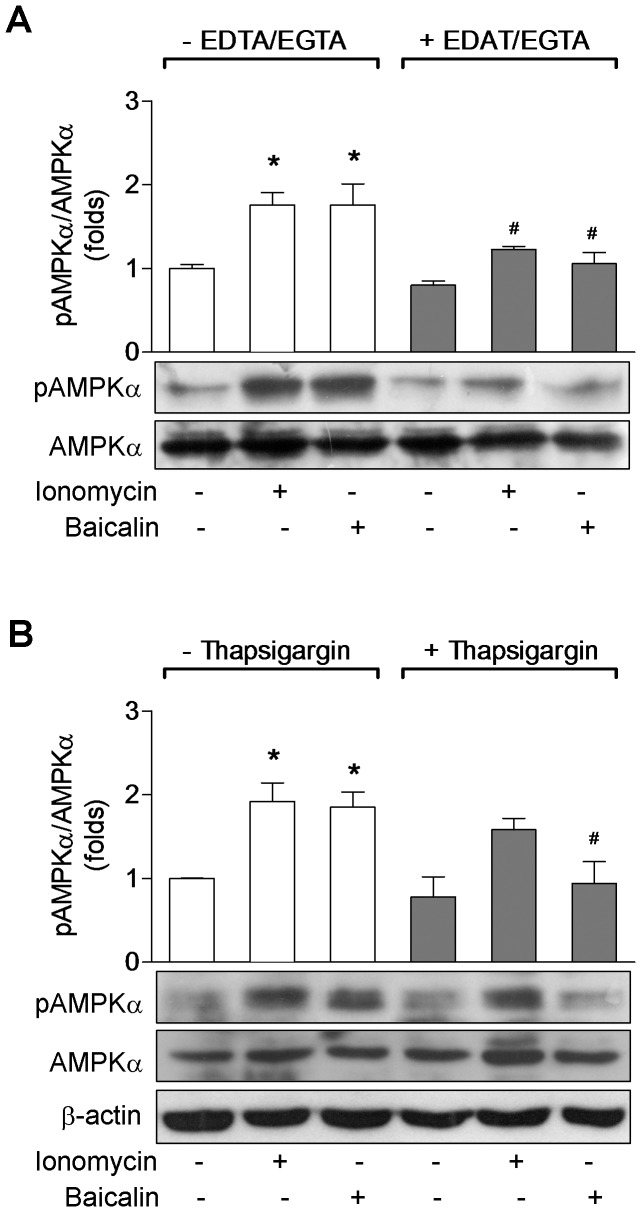
Role of intracellular Ca^2+^ in baicalin-induced AMPKα phosphorylation. HeLa cells were treated with 5 µM baicalin for 1 hr or 1 µM ionomycin for 5 min in control medium (open bars), Ca^2+^-free medium containing Ca^2+^ chelators EDTA and EGTA (solid bars, A), or medium containing 2 µM thapsigarging to deplete intracellular Ca^2+^ stores (solid bars, B). Phosphorylation of AMPKα at Thr-172 (pAMPKα), total AMPKα and β-actin expression were measured by Western blotting. Histograms represent the fold change in the pAMPKα/AMPKα ratio at least three independent experiments. * *p*<0.05 compared to control; ^#^ *p*<0.05 compared to respective treatment group in control medium. Representative Western blots are shown.

To further confirm the role of the intracellular Ca^2+^ in the baicalin-induced AMPK activation, we treated HeLa cells with the Ca^2+^ chelators EDTA and EGTA or with thapsigargin before adding baicalin. We found that either chelating intracellular free Ca^2+^ with 5 mM EDTA and 5 mM EGTA (Figure 4A) or depleting the ER Ca^2+^ by thapsigargin (Figure 4B) significantly reduced the enhanced phosphorylation of AMPKα caused by baicalin, which was correlated with the suppressed [Ca^2+^]_i_ in cells receiving thapsigargin (Figure 3D and E). Interestingly, while chelating intracellular Ca^2+^ prevented ionomycin-induced phosphorylation of AMPKα (Figure 4A), depletion of intracellular stores by thapsigargin did not affect AMPK response to ionomycin (Figure 4B). These data show that intracellular Ca^2+^ is necessary for AMPK activation stimulated by baicalin.

**Figure 5:**
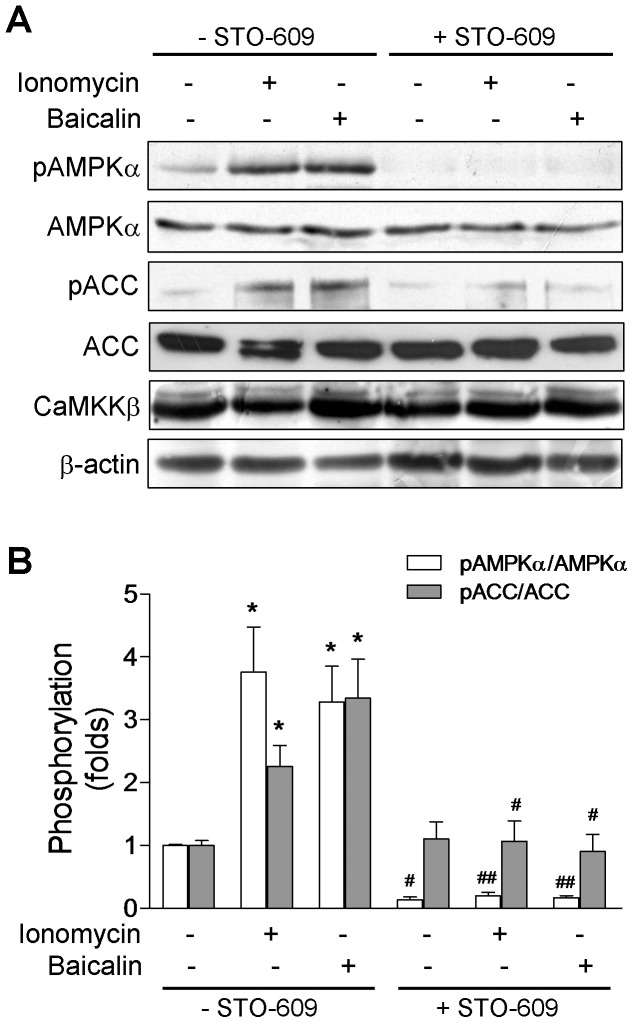
Inhibition of CaMKKβ blocks baicalin-induced AMPKα and ACC phosphorylation in HeLa cells. (A) Representative Western blots showing the effect of the CaMKKβ inhibitor STO-609 on baicalin-induced phosphorylation of AMPKα at Thr-172 (pAMPKα) and phosphorylation of ACC at Ser-79 (pACC) as well as total AMPKα, ACC and CaMKKβ protein expression. Cells were treated with 5 µM baicalin for 1 hr or 1 µM ionomycin for 5 min in the absence or presence of 10 µg/mL STO-609. (B) Histogram represents the fold change in the pAMPKα/AMPKα or pACC/ACC ratio from at least three independent experiments. * *p*<0.05 compared to respective untreated control; ^#^ *p*<0.05 and ^##^ *p*<0.01 compared to respective treatment group in the absence of STO-609.

Because CaMKKβ is activated by Ca^2+^/calmodulin binding, baicalin was able to increase [Ca^2+^]_i_ (Figure 3B and E), and it was important for the AMPK activation (Figure 4), CaMKKβ seemed the probable upstream kinase. CaMKKβ is the predominant isoform regulating AMPK in HeLa cells as demonstrated previously [16] and its expression in HeLa cells is confirmed in the present study by western blotting (Figure 5A). To examine the potential role of the CaMKKβ activation in baicalin-induced AMPK phosphorylation, we treated HeLa cells with STO-609, a selective CaMKKβ inhibitor [15], [30]. We found that pre-treatment with STO-609 completely abolished the phosphorylation of AMPKα and ACC (Figure 5) in response to baicalin or ionomycin, indicating that baicalin stimulated the phosphorylation of AMPKα and ACC in a CaMKKβ-dependent manner.

**Figure 6:**
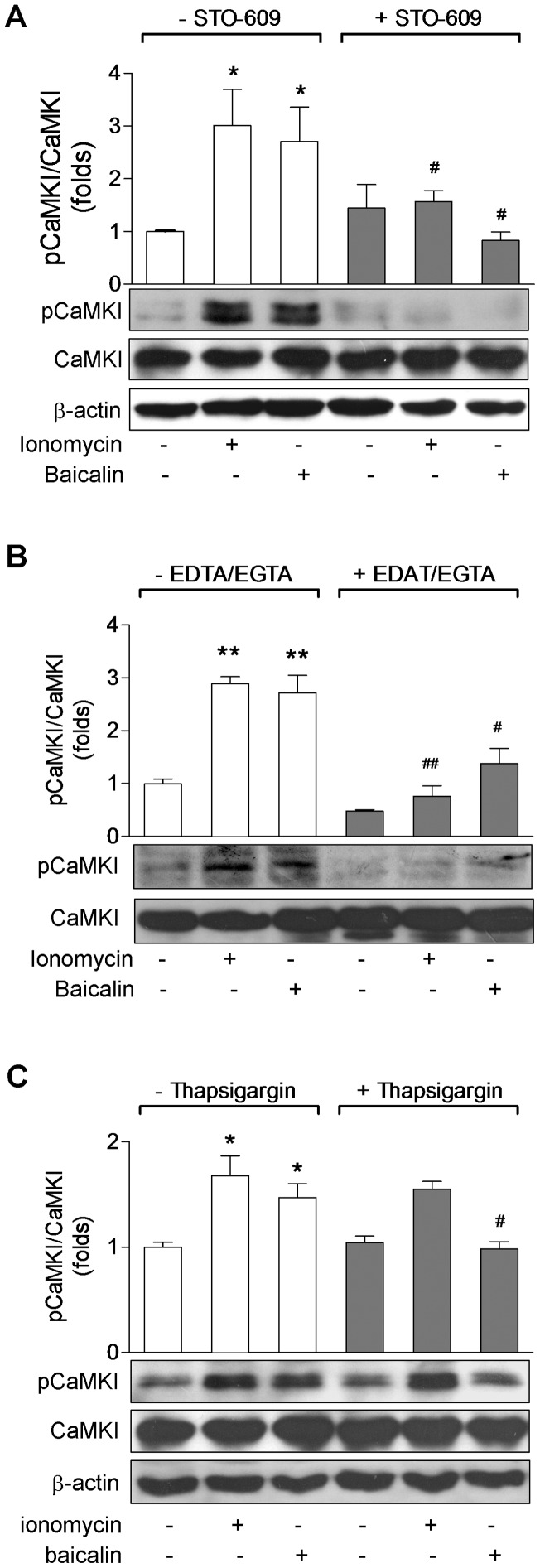
Effects of inhibition of CaMKKβ and intracellular Ca^2+^ on baicalin-induced phosphorylation of CaMKI in HeLa cells. HeLa cells were treated with 5 µM baicalin for 1 hr or 1 µM ionomycin for 5 min in control medium (open bars), or in the presence of 10 µg/mL of the CaMKKβ inhibitor STO-609 (solid bars, A), Ca^2+^-free medium containing Ca^2+^ chelators EDTA and EGTA (solid bars, B), or medium containing 2 µM thapsigarging to deplete intracellular Ca^2+^ stores (solid bars, C). Phosphorylation of CaMKI at Thr-177 (pCaMKI) as well as total CaMKI and β-actin protein expression were measured by Western blotting. Histograms represent the fold change in the pCaMKI/CaMKI ratio from at least three independent experiments. * *p*<0.05 and ** *p*<0.01 compared to control; ^#^ *p*<0.05 and ^##^ *p*<0.01 compared to respective treatment group in control medium. Representative Western blots are shown.

To provide further evidence of CaMKKβ activation in response to baicalin, we determined whether CaMKI, an endogenous substrate of CaMKKβ [31], was phosphorylated at Thr-177. As shown in Figure 6, western blot analysis of total cell lysates using a specific anti-pThr-177 antibody revealed a significant increase in the phosphorylation of CaMKI-Thr-177 (pCaMKI) in the cells treated with baicalin as well as with ionomycin without effect on the total CaMKI protein. The CaMKI response to baicalin or to ionomycin was also completely blocked after inhibition of CaMKKβ by STO-609 (Figure 6A), which is consistent with the inhibition of enhancement of AMPKα phosphorylation caused by baicalin or by ionomycin (Figure 5). Like the effect of intracellular Ca^2+^ on baicalin-induced AMPK activation (Figure 4), baicalin-stimulated CaMKI phosphorylation was also completely abolished after pre-incubation of HeLa cells with EDTA and EGTA (Figure 6B) or with thapsigargin (Figure 6C). Similarly, CaMKI response to ionomycin was completely abolished when pre-incubation of the cells with EDTA and EGTA (Figure 6B), but not with thapsigargin (Figure 6C). Taken together, these data indicate that baicalin-induced activation of AMPK signaling in HeLa cells is likely to be mediated by CaMKKβ.

### Role of CaMKKβ in Baicalin-mediated AMPK Activation in the Presence of LKB1

**Figure 7:**
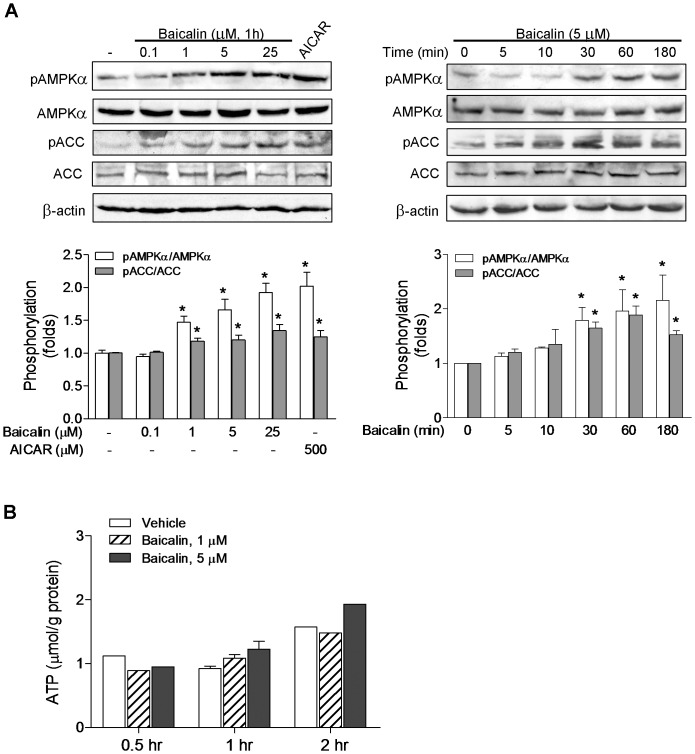
Baicalin increases the phosphorylation of AMPKα and ACC without effect on ATP in HepG2 cells. (A) Levels of AMPKα phosphorylated at Thr-172 (pAMPKα) and of AMPK substrate ACC phosphorylated at Ser-79 (pACC) as well as total AMPKα and ACC were determined by Western blotting. Cells were treated with various concentrations of baicalin or AICAR (500 µM, 2 hr) for the indicated times. Histograms represent the fold change in the pAMPKα/AMPKα or pACC/ACC ratio from at least three independent experiments. (B) Cells were incubated with baicalin for indicated times and ATP levels were measured. All values are the mean ± SE for two-three independent experiments. * *p*<0.05 compared to respective control. Representative Western blots are shown.

**Figure 8:**
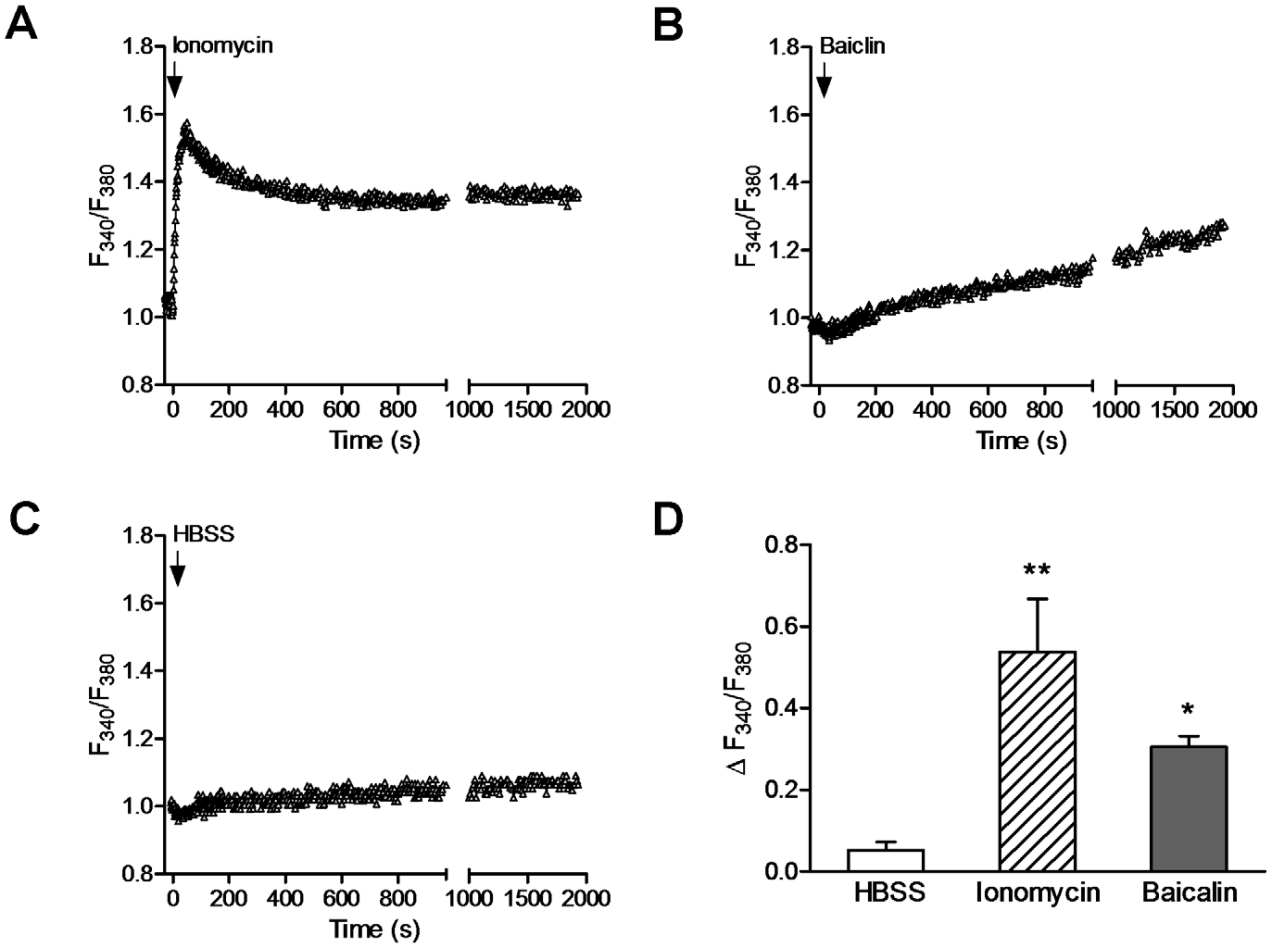
Baicalin increases the intracellular Ca^2+^ levels in HepG2 cells. Traces show the increases in intracellular Ca^2+^ levels in response to application of ionomycin (A) and baicalin (B), as well as application of HBSS solution (C) in HepG2 cells loaded with Ca^2+^ indicator fura 2-AM. Intracellular Ca^2+^ levels were estimated as the ratio of the signals (F_340_/F_380_). (D) Histogram summarizes the changes of intracellular Ca^2+^ levels (ΔF_340_/F_380_) measured after application of HBSS (open bars, n = 3), ionomycin (peak values, hatched bars, n = 3), and baicalin (solid bars, n = 3). * *p*<0.05 and ** *p*<0.01 compared to HBSS control.

**Figure 9:**
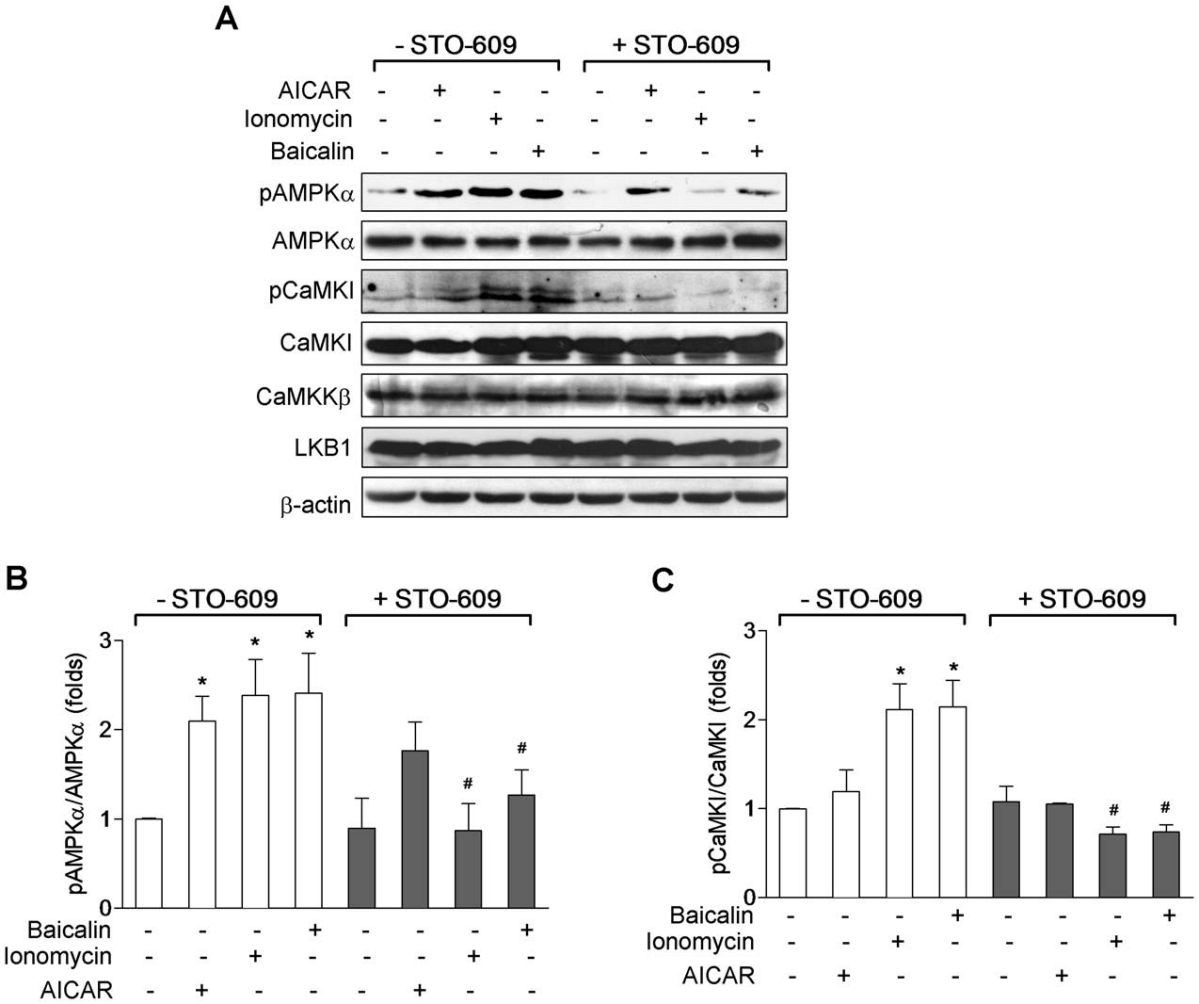
Effects of inhibition of CaMKKβ on baicalin-induced phosphorylation of AMPKα and phosphorylation of CaMKI in HepG2 cells. Cells were treated with 5 µM baicalin for 1 hr or 1 µM ionomycin for 5 min in control medium (open bars) or in the presence of 10 µg/mL of the CaMKKβ inhibitor STO-609 (solid bars). Representative Western blots for phosphorylation of AMPKα at Thr-172 (pAMPKα) and phosphorylation of CaMKI at Thr-177 (pCaMKI), and expression of total AMPKα, CaMKI, CaMKKβ and LKB1 (A). Histograms represent the fold change in the pAMPKα/AMPKα (B) or pCaMKI/CaMKI (C) ratio from at least three independent experiments. * *p*<0.05 compared to control; ^#^ *p*<0.05 compared to respective treatment group in control medium. Representative Western blots are shown.

In order to determine whether CaMKKβ is involved in the regulation of baicalin-mediated AMPK activation in cells that express LKB1, HepG2 cells that express both LKB1 and CaMKKβ were employed for this study. Upon baicalin stimulation, there was a time- and concentration-dependent increase in the phosphorylation of AMPKα and ACC (Figure 7A). [Ca^2+^]_i_ was also measured in HepG2 cells after adding baicalin or ionomycin. As shown in Figure 8, administration of baicalin caused a significant increase in [Ca^2+^]_i_ (ΔF_340_/F_380_∶0.31±0.03, *p*<0.05), which was similar in timing but lower in magnitude to that in HeLa cell, while ionomycin induced a very similar transient increase in [Ca^2+^]_i_ (ΔF_340_/F_380_∶0.53±0.13, *p*<0.01) compared to that in HeLa cells. Concomitant with the increased [Ca^2+^]_i_, an increased phosphorylation of CaMKI (Figure 9A and C) was also observed in HepG2 cells after administration of baicalin or ionomycin. When HepG2 cells were pre-treated with STO-609, the phosphorylation of AMPKα and CaMKI induced by baicalin and ionomycin was markedly inhibited (Figure 9). Like in HeLa cells, baicalin did not significantly alter ATP levels in HepG2 cells (Figure 7B). The protein levels of LKB1 in HepG2 cells (Figure 9A) and of CaMKKβ in HepG2 cells (Figure 9A) as well as in HeLa cells (Figure 5A) were not affected by STO-609 treatment.

In HepG2 cells, AICAR, which did not activate AMPK in HeLa cells (Figure 1A), increased the phosphorylation of AMPKα and ACC (Figure 7A, Figure 9A and B) without significantly altering the phosphorylation of CaMKI (Figure 9A and C). AICAR-induced phosphorylation of AMPKα was not affected by STO-609 pretreatment (Figure 9A and B).

### Baicalin does not Stimulate ROS Production in both HeLa and HepG2 Cells

**Figure 10:**
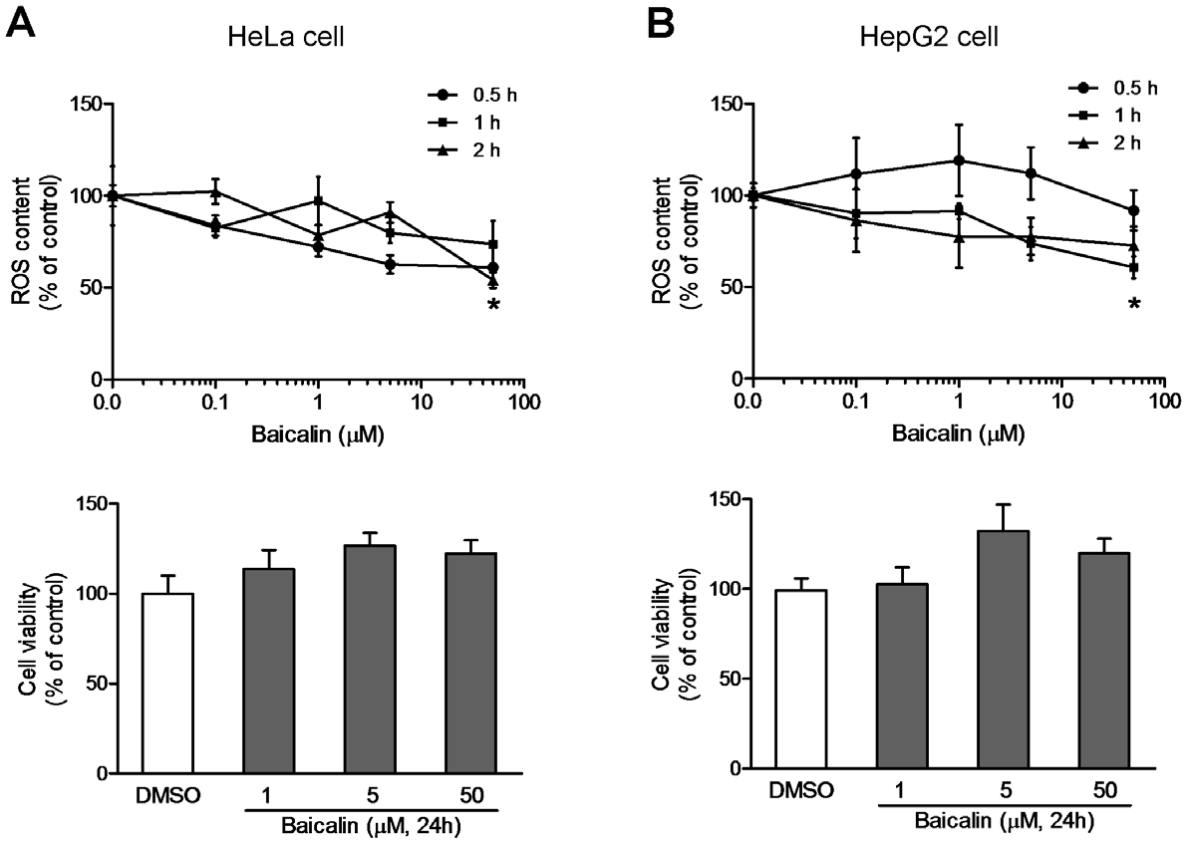
Effects of baicalin on ROS and ATP production. HeLa (A) and HepG2 (B) cells were exposed to various concentrations of baicalin for indicated times. Intracellular ROS production quantified using the fluorescent probe DCFDA (top), and the cell viability was determined by MTT test (bottom). Data were expressed as mean ± SE relative to vehicle control from at least four independent experiments. * *p*<0.05 compared to vehicle control.

Activation of AMPK can also occur in response to oxidant stress and ROS generation [5], [32], [33]. To determine whether endogenous oxidant signals increase in response to baicalin, cellular ROS production was detected by using a cell permeable probe DCFDA dye. As illustrated in Figure 10, treatment of HeLa or HepG2 cells with baicalin (0.1–50 µM) for 0.5, 1 or 2 hr, respectively, did not increase endogenous ROS, in contrast, attenuated the ROS production after incubation of the cells with 50 µM baicalin for 1–2 hr. Thus, baicalin did not appear to activate AMPK via the ROS pathway. Treatment with baicalin (0.1–50 µM) did not show cytotoxic effects in HeLa and HepG2 cells (Figure 10).

### Suppression of CaMKKβ Represses Baicalin-induced Lipid Lowing Effect

**Figure 11:**
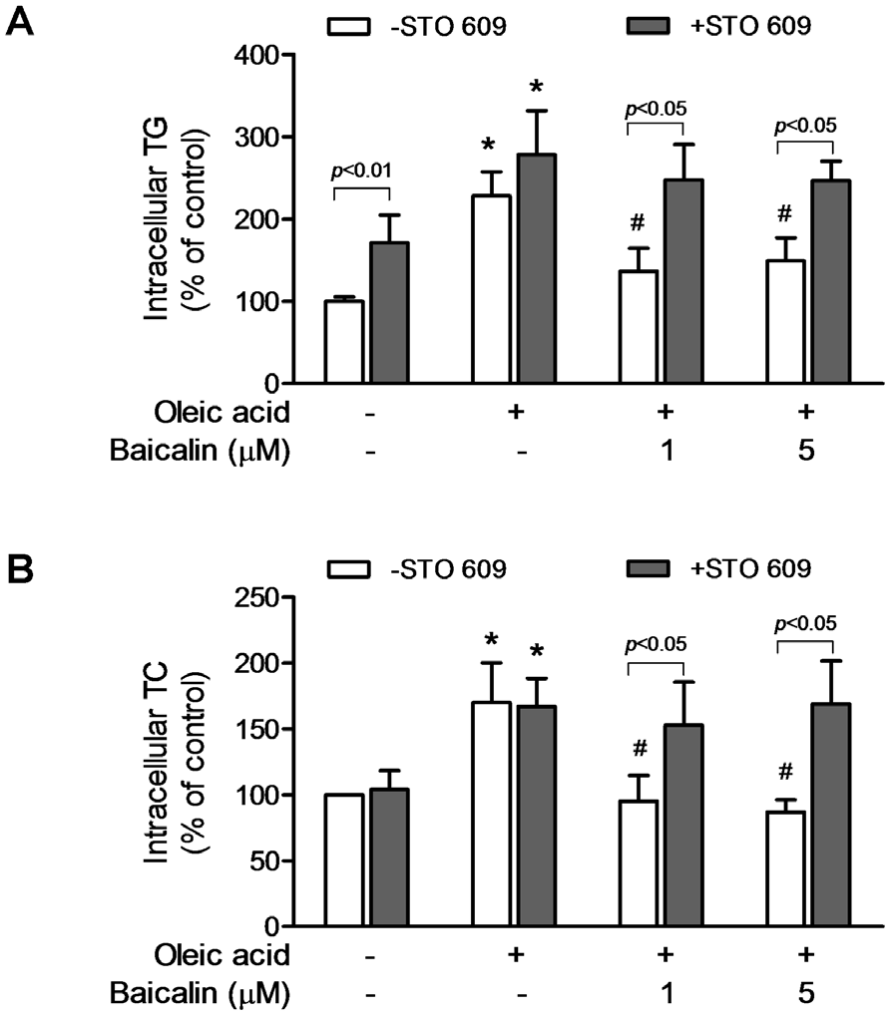
Inhibition of CaMKKβ prevents baicalin-induced reduction in intracellular lipid accumulation in HeLa cells caused by oleic acid. Cells were treated with 1 and 5 µM baicalin in the presence of 0.4 mM oleic acid for 24 hr without (open bars) or with pre-incubated with 10 µg/mL STO-609 (solid bars). The control cells were incubated with 1.76% bovine serum albumin (BSA) for 24 h. Intracellular triglyceride (TG, A) and intracellular cholesterol (TC, B) were measured as described in Material and Methods. The histogram represents the mean of the percentage of the vehicle control ± SE from at least three independent experiments. * *p*<0.05 compared to BSA control; ^#^ *p*<0.05 compared to untreated oleic acid control.

We have reported that one consequence of baicalin-induced AMPK activation is to produce hypolipidemic effect in obesity-related fatty liver diseases [11]. To confirm the role of CaMKKβ/AMPK pathway in baicalin-induced lipid reduction, HeLa cells were incubated in medium containing oleic acid to induce lipid accumulation conditions and the effect of baicalin on intracellular lipid levels in the absence and the presence of STO-609 was examined. After incubation the cells with 0.4 mM oleic acid for 24 hr, cellular triglyceride (TG, Figure 11A) and cholesterol (TC, Figure 11B) content significantly increased compared with the cells grown under normal culture conditions, indicating that the cell model of steatosis was successfully induced by oleic acid. Baicalin at 1 and 5 µM markedly reversed elevated lipid levels caused by oleic acid, consistent with our previous report [11]. When HeLa cells were pre-treated with CaMKKβ inhibitor STO-609, basal TG increased significantly (Figure 11A). Pre-incubation with STO-609 completely blocked baicalin-induced reduction of intracellular lipid accumulation caused by oleic acid (Figure 11A and B), indicating that baicalin reduced intracellular lipid through CaMKKβ-mediated AMPK activation.

## Discussion

Baicalin, one of the major flavonoids in a traditional Chinese herb medicine, *Scutellaria baicalensis*, possesses multiple biochemical and pharmacological activities including antioxidant, anti-inflammatory, antibacterial, immune-stimulating, antiallergic, and antiviral effects [34], [35], [36], which in recent years has gained increasing attention for therapeutic implications for chronic diseases, including dyslipidemia and diabetes [37]. Recently, we have demonstrated that baicalin protected against the development of hepatic steatosis and obesity in rats induced by a long-term HFD and its protective effect is mainly associated AMPK activation and suppression of hepatic fatty acid synthase (FAS) and sterol regulatory element binding protein 1c (SREPB-1c) gene expression [11], [38], however, the underlying mechanism for AMPK activation is unknown. Here, for the first time, we have investigated an important role of a Ca^2+^- and CaMKKβ-mediated pathway in the regulation of baicalin-mediated AMPK activation, and demonstrated that CaMKKβ acts as an upstream kinase for baicalin-mediated AMPK activation.

In two LKB1-deficient cell lines, HeLa and A549 cells, baicalin stimulates AMPK activation and phosphorylates AMPK substance ACC to a similar degree as does in HepG2 cells that express wild-type LKB1. We believe that baicalin-induced AMPK activity is most likely due to the action of another upstream kinase such as CaMKKβ. This conclusion is supported by several previous studies in LKB1-deficient cell lines, such as HeLa and A549 cells, demonstrating that AMPK activity can be stimulated by Ca^2+^ ionophore ionomycin via upstream kinase CaMKKβ [15], [16], [17], [39]. Our data also showed that, in HeLa cell, AMPK can be activated by ionomycin (Figure 4), but not AICAR (Figure 1), which is believed to activate AMPK through the action of LKB1 and an increased AMP:ATP ratio [14], [15], [39]. In addition, pharmacological inhibition of CaMKKβ by its selective inhibitor STO-609 [30] completely abolished ionomycin-, as well as baicalin-induced AMPK activation in HeLa cells (Figure 5). The results further showed that in HepG2 cells, baicalin- and ionomycin-induced AMPK activity, but not AICAR, can also be substantially reduced by STO-609 pretreatment (Figure 9A and B), indicating that baicalin-stimulated AMPK activity can be mediated by CaMKKβ.

Although some studies have shown that baicalin is able to increase [Ca^2+^]_i_ [23], the kinetics of intracellular Ca^2+^ upon stimulation by baicalin is unknown. To determine whether Ca^2+^ signaling could be driving AMPK activation upon stimulation by baicalin we measured [Ca^2+^]_i_ in both HeLa and HepG2 cells with fura2-AM. We found that baicalin caused a slow increase in [Ca^2+^]_i_, and reached to steady-state conditions about 1–2 hr, while ionomycin evoked a rapid transient increase in intracellular Ca^2+^ within 30 sec. The kinetics of [Ca^2+^]_i_ in response to baicalin or ionomycin was not difference between HeLa (Figure 3) and HepG2 (Figure 8) cells. In accordance with their Ca^2+^ signaling, the time course of baicalin-induced increase in phosphorylation of AMPKα and ACC occurred and reached maximal levels within 1 hr and maintained for several hours (Figure 1, Figure 7A), while increases in the phosphorylation of AMPKα and ACC in response to ionomycin occurred within 5 min (Figure 5, Figure 9A and B). The different time-course of AMPK activity induced by baicalin and by ionomycin may imply the different mechanisms for their increasing [Ca^2+^]_i_. We have previously shown that ionomycin increased [Ca^2+^]_i_ globally due to a strong Ca^2+^ influx component [40]. This may explain why elimination of intracellular free Ca^2+^ could significantly prevent AMPK responses to ionomycin, but not by depletion of the intracellular Ca^2+^ stores by thapsigargain (Figure 4B), indicating that ionomycin-caused fast Ca^2+^ influx contributes to its CaMKKβ signaling. Whereas baicalin-induced slow increase in [Ca^2+^]_i_ may involve Ca^2+^ release from the ER since thapsigargin markedly abrogated baicalin-evoked Ca^2+^ signaling (Figure 3D and E), and subsequent AMPK response to baicalin (Figure 4B). Although the precise mechanism by which baicalin increases [Ca^2+^]_i_ still remains unclear, baicalin might induce a progressive Ca^2+^ signaling partially through Ca^2+^ release from the ER that might explain the kinetics of AMPK response to baicalin. A rise in cellular Ca^2+^, along with calmodulin, initiates CaMKKβ signaling pathway leading to AMPK activation [15], [16], [17]. Indeed, our data show that chelation of intracellular free Ca^2+^ by EDTA and EGTA (Figure 4A), or inhibition of CaMKKβ by STO-609 markedly inhibited AMPK activity in response to baicalin. These results are consistent with previous suggestions that CaMKKβ activates AMPK under conditions in which intracellular Ca^2+^ is increased and independently of LKB1 [15], [16], [17].

Activation CaMKKβ also phosphorylates CaMKI [31]. Our data show that enhanced phosphorylation of CaMKI also occurred upon baicalin stimulation, and disappeared by STO-609 or tharpsigargin pretreatment, or after elimination of intracellular free Ca^2+^ with EDTA/EGTA. This represents the activation of CaMKKβ by baicalin.

Activation of AMPK can also occur in response to oxidant stress and ROS generation [5], [32], [33]. Our previous studies [12] have shown that palmitate known as a lipototoxic fatty acid increased the oxidative stress and ROS production, and increased the phosphorylation of AMPK slightly. However, the structurally related flavone lutiolin suppressed the ROS production and further enhanced the phosphorylation of AMPK, led to a reduction of lipid accumulation in HepG2 cells caused by palmitate [12]. To determine whether ROS was involved in baicalin-stimulated AMPK signaling, we detected cellular ROS production in response to baicalin by using a DCFDA dye. The results showed that baicalin did not increase ROS, in contrast, attenuated the ROS production when increased the incubation time. Thus, it is unlikely that ROS signaling contributes to baicalin-stimulated AMPK activation.

Our results also provide evidence that CaMKKβ-mediated AMPK activation could contribute to baicalin lowering effect on intracellular lipid accumulation in HeLa cells caused by oleic acid (Figure 11). AMPK is activated primarily by a stress that depletes cellular ATP or increases ATP consumption, this is amplified by adenylate kinase into a much larger increase in the AMP:ATP ratio [41], [42]. Although we have shown here that there is no significant change in cellular ATP upon baicalin stimulation in HeLa (Figure 1C) and HepG2 (Figure 7B) cells, we cannot at this stage exclude that baicalin could activate AMPK through the AMP/LKB1 pathway for its protective effect on hepatic steatosis suggested by our previous studies [11].

In summary, our findings reveal that baicalin can activate AMPK in the apparent absence of LKB1 partially through Ca^2+^ release from the ER, leading to increases in cytosolic Ca^2+^ that activate the AMPK upstream kinase CaMKKβ, and subsequent AMPK activation. Our study supports the idea that this mechanism might play an important role in cell responses to natural polyphenols, like baicalin, and function in vivo as a protective pathway.

## Materials and Methods

### Materials

Baicalin (HLPC content >95%) and oleic acid were purchased from Sigma. Anti-phospho-AMPKα (Thr 172) and AMPKα antibodies, anti-phospho-ACC (Ser 79) and ACC antibodies, and anti-β-actin antibody were purchased from Cell signaling Technology (MA, UAS). Anti-CaMKKβ, anti-phospho-CaMKI (Thr 177) and CaMKI antibodies were purchased from Santa Cruz Biotechnology, Inc. (Santa Cruz, CA). Anti-mouse and anti-rabbit antibodies conjugated to horseradish peroxidase were obtained from Kirkegaard & Perry Laboratories (Gaithersburg, MD). Minimum Essential medium (MEM), Ham’s F-12 medium, fetal bovine serum, penicillin-streptomycin solution and sodium pyruvate were purchased from Invitrogen. Other chemicals used were fura 2-AM from Molecular Probes. AICA-Riboside (AICAR) and protease inhibitor cocktail set I were purchase from Calbiochem. STO-609 was purchased from Tocris Bioscience. Ionomycin, thapsigargin, and 2′,7′-dichiorofluorescin diacetate (DCFDA) were purchased from Sigma. All other reagents were of analytical grade.

### Cell Culture and Treatments

Human HeLa cells, A549 cells and HepG2 cell were obtained from the American Type Culture Collection (ATCC). HeLa and HepG2 cells were cultured in MEM medium containing 10% (vol/vol) fetal bovine serum, 2 mM L-glutamine, 1 mM sodium pyruvate, 100 U/mL penicillin, 100 µg/mL streptomycin, and 1 mM sodium pyruvate. A549 cells were cultured in Ham’s F-12 medium supplemented with 10% (vol/vol) fetal bovine serum, 100 U/mL penicillin and 100 µg/mL streptomycin. All cell lines were maintained at 37°C in a humidified atmosphere containing 5% CO_2_. Cells were grown to 80% confluence and incubated in serum-free medium overnight before treatments. The cells were exposed to various concentrations of baicalin or to vehicle (DMSO) for the indicated times. The final concentration of DMSO did not exceed 0.1%, which did not affect cell viability or AMPKα phosphorylation. When cells were grown in media supplemented with fatty acids, oleic acid was complexed in MEN containing 1.76% (w/v) bovine serum albumin (BSA), to give a final oleic acid concentration of 0.4 mM.

### Western Blot Analysis

HeLa, A549 or HepG2 cells were cultured in 100-mm culture dishes. The cells used were either under resting conditions or stimulated with various concentrations of baicalin for the indicated times after being serum quiescent for 24 h. After treatment, cells were harvested in a lysis buffer (20 mM Tri, pH 7.5, 150 mM NaCl, 1 mM EDTA, 1 mM EGTA, 1% Triton X-100, 2.5 mM sodium pyrophosphate, 1 mM ß-Glycerolphosphate, 1 mM Na3VO4, 1 µg/mL Leupeptin, 1 mM PMSF). Samples were sonicated three times for 5 s with 15 s breaks between cycles, and centrifuged at 16000×*g* for 60 min at 4°C. Protein concentrations of the supernatants were determined with a protein assay kit (Bio-Rad). Equal amounts of total cellular proteins were resolved by 10% SDS-PAGE transferred onto polyvinylidene difluoride membranes (Amersham Biosciences) and then probed with primary antibody followed by secondary antibody conjugated with horseradish peroxidase. The immunocomplexes were visualized with enhanced chemiluminescence kits (Amersham Biosciences).

### Measurement of Intracellular Ca^2+^ Levels

HeLa or HepG2 cells were loaded with 5 µM fura 2-AM in Hanks balanced salt solution (HBSS, containing 5.3 mM KCl, 0.3 mM Na_2_HPO_4_, 0.4 mM KH_2_PO_4_, 4.2 mM NaHCO_3_, 1 mM CaCl_2_, 0.5 mM MgCl_2_, 0.4 mM MgSO_4_, 137 mM NaCl, and 5.6 mM D-glucose, pH 7.4) for 30 min at room temperature. After removing the loading buffer, cells were washed with HBSS, incubated with 100 µL HBSS, and excited alternately at 340 and 380 nm using Synergy™ 2 microplate reader (BioTek Instruments, Inc.). The emission signals were collected at 525 nm and stored on a computer. The intracellular Ca^2+^ level were estimated as the ratio of the signals (F_340_/F_380_). Baicalin or ionomycin was acutely applied into the well by a Synergy™ 2 microplate reader (BioTek Instruments, Inc.). Assays were performed in triplicate.

### ATP Measurement

Cellular ATP levels were measured using a firefly luciferase-based ATP assay kit (Beyotime, China) according to the manufacturer’s instructions. ATP contents are expressed as micromoles per gram of protein.

### Measurement of Reactive Oxygen Species (ROS)

Cellular ROS levels were measured using a cell permeable probe DCFDA (Sigma). The cells were loaded with 10 µM DCFDA in PBS for 30 min. After washing the cells with PBS twice, fluorescence was measured by Synergy™ 2 microplate reader (BioTek Instruments, Inc.) at excitation of 485 nm and emission of 525 nm. All the readings were normalized to protein levels (mg/mL) by the Bradford assay. Data are represented as percentages of control cells.

### Cell Viability Assay

Cell viability was assessed with the MTT assay performed according to the manufacturer’s suggestions (Sigma). The cells were grown in 96-well plates at a density of 1×10^4^ cells per well with 100 µL culture medium. After cells were attached, the medium was refreshed with different concentrations of baicalin (0.1–50 µM). For control group, the same concentration of vehicle was added to the medium. After being cultured for 24 h, the cells were incubated with MTT for another 4 h at 37°C. Subsequently, the medium was removed and DMSO was added to each well. The absorbance of the samples was measured at 570 nm using a Synergy™ 2 microplate reader (BioTek Instruments, Inc.). All experiments were performed independently in triplicate.

### Determination of Intracellular Lipid Contents

Intracellular triglyceride (TG) and cholesterol (TC) contents were determined in cell lysates by an enzymatic colorimetric method using a commercially available kit (Shanghai Mind Bioengineering Co, China) and normalized by protein content as previously described. Data are represented as percentages of control cells.

### Statistical Analysis

All data are presented as mean ± SEM. Student’s test (unpaired) was used to determine the statistical significance (*P*<0.05) of obtained data.
